# Digit Ratio (2D:4D) and Handgrip Strength in Hani Ethnicity

**DOI:** 10.1371/journal.pone.0077958

**Published:** 2013-10-30

**Authors:** Dapeng Zhao, Keli Yu, Xinghua Zhang, Lianbin Zheng

**Affiliations:** 1 Tianjin Key Laboratory of Animal and Plant Resistance, College of Life Sciences, Tianjin Normal University, Tianjin, China; 2 College of Life Sciences, Northwest University, Xi’an, China; University of Goettingen, Germany

## Abstract

**Introduction:**

The ratio of the length of the second finger to the fourth finger (2D:4D) in humans is considered as a putative marker of prenatal exposure to testosterone, and has been progressively adopted as one useful tool to evaluate the effect of prenatal hormones in some traits such as physical ability. Handgrip strength is one authentic measure of physical ability and is generally used on the anthropological research within an evolutionary viewpoint.

**Methods:**

Here we present the first evidence on 2D:4D and handgrip strength on adult participants of Hani ethnicity and explore the relationship between digit ratio (2D:4D) and handgrip strength. We examined 2D:4D and handgrip strength of 80 males and 60 females at Bubeng village, in the Yunnan province of China.

**Results:**

The mean 2D:4D in females was higher than that in males for each hand. Females showed significantly higher 2D:4D than males in the right hand rather than in the left hand. Males displayed significantly higher handgrip strength than females for both hands. Handgrip strength decreased with age for both sexes. A significant negative correlation between 2D:4D and handgrip strength was found in the right hand of males.

**Conclusion:**

The relationship between 2D:4D and handgrip strength may be attributed to evolutionary drive of sexual selection operating on fetal programming.

## Introduction

In humans, the ratio of the length of the second finger to the fourth finger (2D:4D) is sexually dimorphic [1], [2], in which females have a higher mean 2D:4D than males [3]–[8]. Sex difference on 2D:4D is generally fixed in utero [9], [10] and then present in newborns [11]. Individual 2D:4D remains relatively stable over the lifespan [12]–[15].

The 2D:4D has been linked with prenatal hormone exposure in humans [16]–[20] as well as other mammals [21], and has been found to be related with diverse physical abilities in humans [22]–[28]. Hönekopp and Schuster [29] made a meta-analysis and found that athletic prowess is negatively correlated with 2D:4D of each hand for both sexes.

Handgrip strength, one authentic measure of physical ability, is generally adopted on the anthropological research within an evolutionary viewpoint [30]. Increasing evidences show that handgrip strength is highly sexually dimorphic [31], [32]. Current evidences on the relationship between 2D:4D and handgrip strength is limited and has yielded inconsistent results. Fink et al. [22] examined males of two ethnicities (Caucasian and Mizos) and found a significantly negative correlation between 2D:4D and handgrip strength. Van Anders [33] considered females without controlling for ethnicity and found no significant correlation between 2D:4D and handgrip strength. Gallup et al. [30] focused on both sexes without controlling for ethnicity and did not find a significant correlation between 2D:4D and handgrip strength. Zhao et al. [8] for the first time investigated both sexes in the same ethnicity (Han ethnicity) and brought up a sex-specific relationship between 2D:4D and handgrip strength, i.e. there is a significant negative correlation between right 2D:4D and handgrip strength in males but not in females. Hone and McCullough [34] then found a similar relationship among college students with controlling for ethnicity. However, further validations with more investigations are required on whether such relationship between 2D:4D and handgrip strength is common for any ethnicity in humans.

Given the above background, this study presents first evidence on 2D:4D and handgrip strength for both sexes of Hani ethnicity. Hani ethnicity now is mainly widespread in China, Laos, Vietnam and Burma. Over ninety percent of the Hani people live in Yunnan province of China. In China, there are totally about 1.65 million people of Hani ethnicity (data on the 6th national census of Chinese population: www.stats.gov.cn). Han is the largest ethnic group [8] whereas Hani is considered as an ethnic minority in China. The Hani ethnicity shares the same origin with the Yi and Lahu ethnic groups. Based on Chinese historical records, they all evolved from the ancient Qiang people. Currently, the information on 2D:4D and handgrip strength in the Hani ethnicity is absent. The main purposes in the present study related to Hani ethnicity are to 1) identify whether there is sexual dimorphism in both 2D:4D and handgrip strength; 2) investigate how age influence 2D:4D and handgrip strength for both sexes; 3) explore the relationship between 2D:4D and handgrip strength. We hypothesized that a significant negative correlation between right 2D:4D and handgrip strength in males but not in females in Hani ethnicity based on previous related findings [8], [22], [34].

## Materials and Methods

### Study Site and Samples

This study was conducted at the Bubeng village, which is located at the Xishuangbanna National Nature Reserve in the Yunnan province of China. The participants of Hani ethnicity included 140 adults (80 males and 60 females) in this village (Table 1). All participants were right-handed, healthy without any physical damage on their fingers and arms.

**Table 1:** Descriptive statistics of participants.

	Males (N = 80)			Females (N = 60)		
	Mean	SD	Range	Mean	SD	Range
Age (years)	37.80	12.72	19–73	40.28	14.17	19–77
Left-hand 2D:4D	0.9420	0.0501	0.8495–1.1461	0.9553	0.0304	0.8982–1.0379
Right-hand 2D:4D	0.9318	0.0433	0.8355–1.1218	0.9523	0.0370	0.8977–1.0521
Right-left 2D:4D	−0.0102	0.0542	−0.2343–0.1233	−0.0030	0.0477	−0.1258–0.0967
Left-hand grip strength (kg)	40.45	6.62	24.60–59.40	23.69	5.46	12.60–40.30
Right-hand grip strength (kg)	42.94	6.91	26.90–64.70	26.28	5.15	14.50–40.50

### Ethical Statements

This study was approved by the ethics committee of Xishuangbanna National Nature Reserve. Written permission of the reserve authorities as well as Bubeng villagers to conduct this research was obtained before the onset of the present study.

### Data Collection

Participants put their hands on a flat surface of a desk and straighten their fingers with the palm facing upwards. Then we took photos for each hand respectively and used Photoshop to measure both hands in inches from the tip of the finger to the center of the digit crease proximal to the palm in order to obtain more accurate measurement [34]–[36]. Length measures were made twice for each finger on both hands [8]. Re-measurement reliability was high for the first and second 2D:4D (intraclass correlation coefficients, r_1_, left-hand 2D:4D r_1_ = 0.980, F (1,139) = 100.416, *p*<0.001; right-hand 2D:4D r_1_ = 0.976, F (1,139) = 83.775, *p*<0.001). Then we calculated the mean 2D:4D (Table 1). In addition, the right-left 2D:4D, termed directional asymmetry, was calculated based on the formula: right-left 2D:4D = right 2D:4D - left 2D:4D [37]. The positive value showed a more feminized pattern of digit ratio in the right hand whereas the negative value showed the opposite [8], [38], [39].

We used a dynamometer (Xiangshan EH101 electronic dynamometer, made in China) to measure handgrip strength in kilograms force for each hand. Participants were measured three times for each hand, with an interval of five minutes between each trial. We recorded the maximum strength of the three trials for each hand for the following analysis [8].

### Data Analysis

The independent-samples t test was used to examine sex difference on 2D:4D, right-left 2D:4D and handgrip strength. The Pearson correlation coefficient test was used to explore the relationship between measures. We analyzed the difference between two independent correlation coefficients by the computer software available from the website http://quantpsy.org. We adopted the SPSS 16.0 to conduct all the analyses, with a significance level of p≤0.05.

## Results

### Sex Difference

The mean 2D:4D in females was higher than that in males for both hands (Table 1). Females showed significantly higher 2D:4D than males in the right hand (t = −2.946, *p* = 0.004) but not in the left hand (t = −1.827, *p* = 0.070). There was no significant sex difference on right-left 2D:4D (t = −0.813, *p* = 0.417). Males showed significantly higher handgrip strength than females in the left hand (t_ = _15.964, *p*<0.001) as well as in the right hand (t_ = _15.684, *p*<0.001).

### Age Effect

We found no significant correlation between age and 2D:4D for each sex (males: r = −0.008, *p* = 0.942 for left hand; r = 0.054, *p* = 0.635 for right hand; females: r = −0.107, p = 0.416 for left hand; r = −0.029, *p* = 0.829 for right hand). We also found no significant correlation between age and right-left 2D:4D for each sex (males: r = 0.051, *p* = 0.655; females: r = 0.046, p = 0.726).

**Figure 1:**
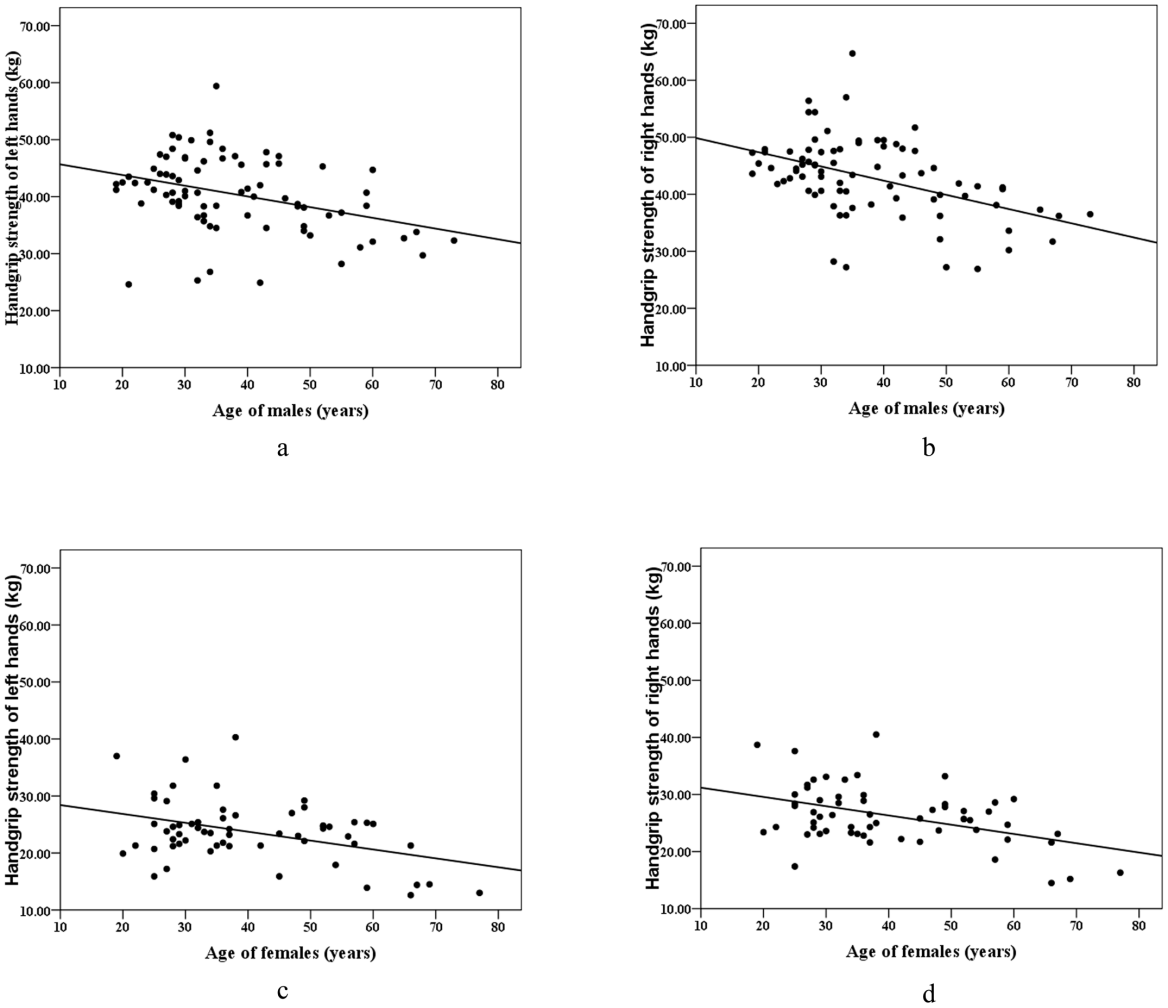
Correlation between age and handgrip strength (kg). a) males, left hands; b) males, right hands; c) females, left hands; d) females, right hands.

A significant negative correlation was found between age and handgrip strength for each sex (males: r = −0.361, *p*<0.001 for left hand; r = −0.459, *p*<0.001 for right hand; females: r = −0.405, *p* = 0.001 for left hand; r = −0.447, *p*<0.001 for right hand) (Figure 1).

### Relationship between 2D:4D and Handgrip Strength

Given the significant age effect on handgrip strength, we made partial correlation analysis and found a significant negative correlation between 2D:4D and handgrip strength for males in the right hand rather than in the left hand (left hand: r = −0.147, *p* = 0.195; right hand: r = −0.242, *p* = 0.032). We found no significant correlation between 2D:4D and handgrip strength in females for both hands (left hand: r = −0.195, *p* = 0.140; right hand: r = −0.185, *p* = 0.161). No significant correlation was found between right-left 2D:4D and right handgrip strength for each sex (males: r = −0.139, *p* = 0.221; females: r = −0.079, p = 0.550).

We found that correlation between 2D:4D and handgrip strength for the right hand was not significantly different from that correlation for the left hand in both sexes (males: z = 0.613, *p* = 0.540; females: z = −0.055, *p* = 0.956). Similarly, correlation between 2D:4D and handgrip strength in males was not significantly different from that correlation in females for each hand respectively (left hand: z = 0.283, *p* = 0.771; right hand: z = −0.342, *p* = 0.732).

## Discussion

To the best of our knowledge, the present study for the first time presents information on 2D:4D and handgrip strength of both sexes in Hani ethnicity. The main findings are 1) the mean 2D:4D in females was higher than that in males for both hands, which supports the related finding on East-Asian 2D:4D [2]; 2) females showed significantly higher 2D:4D than males in the right hand but not in the left hand; 3) no significant sex difference was found on right-left 2D:4D; 4) a significant sex difference was found on handgrip strength, which may be due to some sex-specific traits [32], [40], [41], [42]; 5) handgrip strength decreased with age for both sexes, which is in accord with many previous studies [8], [43]–[46]; 6) as expected, a significant negative correlation between 2D:4D and handgrip strength was only found in the right hand of males.

2D:4D in the right hand is supposed to be more powerful than that in the left hand when predicting anthropic morphology and behavior in that the right hand displays prenatal testosterone levels more exact than the left hand [1], [2], [47]–[50]. Two main contentions were considered to support this view. Firstly, sex difference on 2D:4D in the right hand is more obvious than that in the left hand [6], [48], [49]. Secondly, 2D:4D in the right hand show stronger correlation with predicted variables than that in the left hand [1]. For Hani ethnicity, we found 1) females showed significantly higher 2D:4D than males in the right hand rather than in the left hand; 2) there was a significant negative correlation between 2D:4D and handgrip strength for males in the right hand but not in the left hand. The findings in Hani ethnicity lend further support to the notion that 2D:4D in the right hand is a better index of postnatal morphology and behavior than 2D:4D in the left hand [2].

Sex hormones during prenatal periods of individual development influence sexual differentiation in human behavior [51]–[53]. Our data in Hani ethnicity found that there was a significant negative correlation between 2D:4D and handgrip strength in males rather than in females, which to some extent suggests that prenatal exposure to testosterone is associated with handgrip strength only in males. This finding is consistent with two previous studies [8], [34]. Such specific correlation was also found between 2D:4D and other physical ability such as rowing ergometer performance [23] although it does not back up the finding of Hönekopp and Schuster [29] that athletic power is negatively correlated with 2D:4D for each sex. It may be due to a potential stronger relationship between prenatal androgen exposure and the generation of muscular strength as well as cardiovascular system in males whereas such link is absent in females under the evolutionary drive of sexual selection [22], [23], [54].

All in all, this is the first study on 2D:4D and handgrip strength of both sexes in Hani ethnicity. Sexual dimorphism in both 2D:4D and handgrip strength were found in Hani ethnicity. It further broaden our knowledge on digit ratio among different ethnic groups and strengthen the viewpoint that sexual dimorphism of 2D:4D is universal in humans. For Hani ethnicity, a significant negative correlation between right 2D:4D and handgrip strength was found in males but not in females, which may attribute to the evolutionary adaption of sexual selection. It should be noted that hand preference influence 2D:4D and handgrip strength [37], [55], [56] whereas in the present study all participants were right-handed and the effect of hand preference is still unclear in Hani ethnicity. Therefore it is required to examine this potential factor in the future research.
